# Utilization of Real-World Data in the Intervention to Reduce Early Peanut Allergy in Children (iREACH) Trial

**DOI:** 10.23889/ijpds.v9i5.2859

**Published:** 2024-09-18

**Authors:** Lucy Bilaver, Jialing Jiang, Ruchi Gupta

**Affiliations:** 1 Northwestern University Feinberg School of Medicine; 2 Ann & Robert H. Lurie Children's Hospital of Chicago

## Objective

To demonstrate the use of linked, real-world data in the context of a pediatric practice-based clinical trial.

## Approach

The Intervention to Reduce Early Peanut Allergy in Children (iREACH) is a two-arm, cluster randomized, controlled trial. The intervention included clinician education and clinical decision support to increase pediatric clinician adherence to the NIAID’s 2017 Prevention of Peanut Allergy guidelines during infants’ 4- and 6-month well child care visits. Electronic health record (EHR) data were shared with the data coordinating center under a Health Insurance Portability and Accountability Act (HIPAA) waiver. All caregivers of eligible infants were invited to complete two surveys via REDCap, an online survey platform. The consents included an optional HIPAA authorization to link EHR data with survey responses and authorization to collect and link administrative data from the Special Supplemental Nutrition Program for Women, Infants, and Children (WIC) program, if applicable.

## Results

Overall, 30 Illinois pediatric practices participated. EHR data was collected for 18,480 infants of whom 4,331 had any caregiver survey data (23.4% response rate). Permission to link survey responses to EHR data was obtained for 3,950 infants among those with any survey data (91.2%). Consent for linking WIC data with study data was obtained for 229 infants and 194 mothers.

## Conclusions

Authorization for data linkage in the consent forms allowed investigators to amass data to compare caregiver and clinician responses, generate hypotheses, and propose future research outside the scope of the main trial.

## Implications

Multiple real-world data sources can be effectively combined to extend the findings of clinical trials.

